# Population prevalence and correlates of prolonged and shortened QTc intervals in a nationwide survey of adults in China: a report from Chinese arrhythmia epidemiology cross-sectional study

**DOI:** 10.3389/fcvm.2025.1555512

**Published:** 2025-07-31

**Authors:** Li-Guo Tan, Jing-Xuan Liu, Fei Guo, Jing Lin, Ran Xiong, Hao Fu, Hao-Ming You, Liu He, Shi-Jun Xia, Xin Du, Jian-Zeng Dong

**Affiliations:** ^1^ Department of Cardiology, Beijing Anzhen Hospital, Capital Medical University, Beijing, China; ^2^ Department of Cardiology, Shiyan Renmin Hospital, Hubei University of Medicine, Shiyan, Hubei, China

**Keywords:** QTc intervals, prolonged QTc intervals, shortened QTc intervals, nationwide survey, QTc abnormalities

## Abstract

**Objective:**

QT interval irregularities correlate with severe arrhythmias and sudden cardiac death. However, epidemiological data on QT intervals in Chinese adults are lacking. This study aimed to elucidate the distribution of the QT interval (QTc) patterns in Chinese adults and the risk factors associated with a prolonged QTc interval and shortened QTc interval.

**Methods:**

This study was based on data from the Chinese Arrhythmia Epidemiology Cross-Sectional Study conducted in seven provinces of China between 2014 and 2016. A total of 42,031 Chinese adults (age ≥ 45 years) were included in the study, and body surface electrocardiograph (ECG) QTc and other indices were systematically analyzed retrospectively among the participants.

**Results:**

The mean QTc interval in Chinese adults was 429.4 ± 25.1 ms (men: 429.7 ± 25.2 ms; women: 430.0 ± 22.0 ms; *P* = 0.97). The 2.5th and 97.5th percentile QTc intervals were 384 and 480 ms, respectively. The prevalence of a long QTc interval (QTc > 440 ms) and a very long QTc interval (QTc > 500 ms) in Chinese adults was 32.64% and 0.60%, respectively. Multifactorial logistic regression analysis found that Han Chinese ethnicity, rural residence, hypertension and health insurance were independently associated with an increased risk of long QTc intervals (all *P* < 0.05). The prevalence of short QT intervals was 4.87% (American Heart Association criteria), 1.84% (European Society of Cardiology criteria), and 0.02% (heart rhythm criteria). Individuals with QTc < 320 ms were not observed in this study. Abnormal renal function (eGFR (estimated glomerular filtration rate) <90 ml/min/m^2^) was independently linked to an elevated risk of short QTc intervals; hypertension was strongly associated with reduced short QTc intervals.

**Conclusion:**

This study provides epidemiological data on the distribution of QT intervals in Chinese adults. Range of the normal QTc interval in Chinese adults is 384–480 ms. The QTc interval is longer in Chinese adults of Han Chinese ethnicity than in those of non-Han Chinese ethnicity. Hypertension is closely associated with a long and short QTc interva, which should be considered when administering medications to hypertensive patients in clinical practice. No individuals with a QTc interval ≤320 ms were observed in this study, indicating that short QT syndrome may be very rare in the Chinese adult population.

## 1 Introduction

The QT interval represents the combined time span of ventricular depolarization and repolarization and is an important electrocardiographic index (1, 2). Because it is easy to obtain and is strongly associated with malignant arrhythmias, cardiovascular events, and all-cause mortality (3–5), the QT interval is presently regarded as one of the potentially significant vital signs.

The QT interval is strongly affected by heart rate. Numerous formulas have been formulated to account for the impact of heart rate on the QT interval, with the Bazett formula representing the most frequently applied method. The length of the QTc interval is affected by an intricate interplay between genetic and environmental factors. Differences in race, geography, comorbidities, lifestyle, drug exposure, and study population yield different QTc values (6–8). For example, QTc interval was significantly shorter in British African-Caribbean individuals than in Caucasian individuals and that the QTc interval exhibited a significant prolongation in patients with HIV compared to healthy controls (9, 10). Therefore, it is not reasonable to establish a uniform QTc standard.

QT interval abnormalities include either a prolonged QT interval or a shortened QT interval. Previous studies have focused primarily on QT prolongation, and a large number of researches have indicated that prolonged QT intervals are strongly associated with malignant arrhythmias, cardiovascular death, and all-cause mortality (3, 4). Short QT syndrome is a cardiac ion channel disease, which characterized by accelerated repolarization and manifests on ECG as a shortened QT interval (QTc < 300 ms) (11, 12). Studies have shown that QT interval shortening also increases the risk of adverse outcomes, including malignant arrhythmias and sudden death (5). The short QT interval overlaps to some extent with the QT interval in short QT syndrome (SQRS) (13). Gaita et al. followed 53 patients with a mean QTc value of 314 ms for 64 months and found that 32% experienced sudden death (14). However, genetic mutation detection found that only 23% of the prevalent patients had mutations in SQTS-related genes, suggesting that this borderline shortening of QT may also be strongly associated with adverse events such as sudden death.

The definition of normal and abnormal QTc ranges in the general population is extremely important, especially for pharmaceutical companies and clinical work, where it has a huge impact. Furthermore, overestimation of abnormal QTc intervals will lead to waste of medical resources and increase unnecessary examinations. Underestimating abnormal QTc interval leads to an inadequate assessment of disease risk.

China is a multiethnic country with a vast territory and a large population. However, the QTc criteria currently used in clinical work in China are derived from data from Western population, and there exists a scarcity of large-scale epidemiological data regarding the QTc interval in the Chinese population.

This study uses data from a representative community-based survey of Chinese individuals and aims to provide reliable contemporary data to establish the QTc interval distribution in Chinese adults (15), to evaluate the prevalence of long and short QTc intervals and associated risk factors, and to address the data gap in this field in China.

## 2 Methods

### 2.1 Ethics

The study protocol was approved by the Clinical Research Ethics Committee of Beijing Anzhen Hospital. Each participant provided written informed consent. The data, analytical methods, and materials employed in the study will be accessible to investigators for replicating the findings, upon submission of a formal request to the corresponding author.

### 2.2 Study subjects

The methods of this study have already been described in a prior publication (15, 16). A total of 64,893 individuals who were invited to participate across 39 locations spanning seven regions, 47,841 (age ≥ 45 years) completed the survey. In the first phase, regions including Beijing, Guangdong, Henan, Jilin, Jiangxi, the Xinjiang Uyghur Autonomous Region, Yunnan, and Zhejiang were randomly selected to represent China's seven geographical zones (Northeast, Northwest, North, Central, East, South, and Southwest China).

In the second phase, we randomly selected 4,000 rural residents (1–13 representative villages) residents and 4,000 urban residents (from one to three representatives of communities in a capital city), each having resided in the area for more than 6 months, from each city or province. In the third phase, door-to-door visits were conducted in selected communities and villages up to three times on different days to invite eligible individuals to participate. No patients or members of the public were involved in the design or analysis of this study. Each participant provided written informed consent.

**Figure 1:**
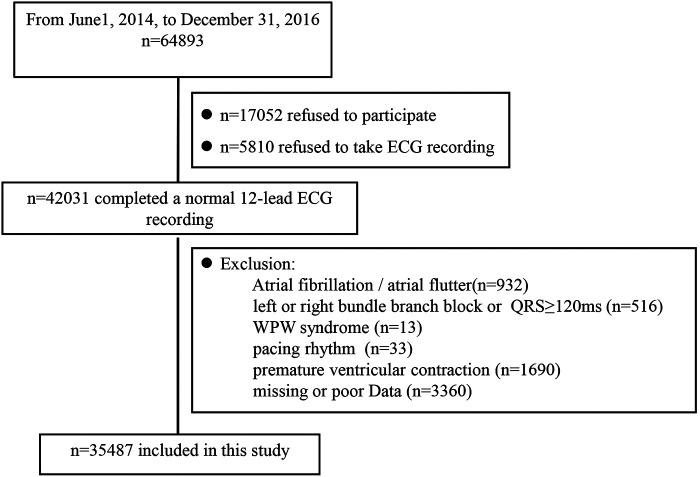
Overview of the study population. ECG, electrocardiograph.

From among the 42,031 who completed a 12-lead ECG recording, we excluded those who had poor ECG data (*n* = 3,360) and ECG indicating various arrhythmias (932 with atrial fibrillation or atrial flutter, 516 with QRS duration of 120 ms or longer. complete left or right bundle branch block, 13 with WPW syndrome, 33 with pacing rhythm, 1,690 with premature ventricular contraction); finally, 35,487 participants were included in our survey. A flow chart describing the patient selection process is reported in Figure 1.

### 2.3 Data collection and measurements

All eligible participants were invited to a designated survey location to complete a structured, face-to-face interview conducted by trained research personnel using a standardized questionnaire, followed by a physical examination and laboratory assessments. Data were obtained regarding socio-demography (age, sex, marital status, education level, household income and medical insurance coverage) and medical history of any symptoms, diagnosis and treatment of hypertension, diabetes mellitus, dyslipidemia, coronary heart disease (CHD), stroke/transient ischemic attack (TIA) and lifestyle factors, including diet, smoking, alcohol consumption (days per week) and physical activity, according to the modified Global Physical Activity Questionnaire (17).

A 5 ml blood sample was collected from the antecubital vein of each participant in the morning following an overnight fast of at least 8 h. The samples were analyzed centrally for blood glucose, creatinine, total cholesterol (TC), low-density lipoprotein cholesterol (LDL-C), and triglycerides at GuangZhou Kingmed Testing Science & Technology, a laboratory accredited by the College of American Pathologists.

A MAC 5500 (GE Healthcare; Little Chalfont, Buckinghamshire, UK) was used by well-trained cardiologists to perform 12-lead ECGs (rest 10 s) for all participants, and the results were analyzed automatically through the MUSE Cardiology Information System, version 7.0.0 (GE Healthcare). Bazett's formula was then used to correct QT intervals for heart rate.

All ECGs with QT ≥ 500 ms or QTc ≥ 500 ms and all ECGs with QT < 360 ms or QTc < 360 ms, respectively, were evaluated by an electrophysiologist to verify the accuracy of the QT measurements.

### 2.4 Definitions

Smoking status was defined as nonsmoker, former smoker (≥1 year) or current smoker according to self-report. Habitual drinking was defined as self-reported alcohol use ≥5 days per week in the previous year. Manual labor included farmers, fisheries, factory workers and salespeople. Hypertension was defined as measured systolic blood pressure (SBP) ≥140 mm Hg and/or diastolic blood pressure (DBP) ≥90 mm Hg, taking antihypertensive medication or self-report of a diagnosis of hypertension given by a physician. Diabetes mellitus was defined as a fasting blood glucose ≥7.0 mmol/L, self-report of a previous test with postprandial blood glucose ≥11.1 mmol/L or HbA1c ≥ 7%, taking antidiabetic medicines or self-report of a diagnosis given by a physician. Dyslipidemia was defined as TC ≥ 240 mg/dl, LDL-C ≥ 160 mg/dl, taking a statin or other lipid-lowering agent or self-report of the diagnosis given by a physician. Obesity was defined as body mass index (BMI) ≥ 28 kg/m^2^. CHD was defined as any history of myocardial infarction (MI), percutaneous coronary intervention or coronary artery bypass grafting. Stroke was defined as a diagnosis given by a physician or self-report of any acute-onset, TIA or permanent neurological deficit. Sedentary lifestyle was defined according to the definition used in the global surveillance of physical activity levels of adults: 30 min of moderate-intensity leisure-time physical activity ≥5 days per week or 20 min of vigorous-intensity leisure-time physical activity ≥3 days every week (18).

### 2.5 QT interval

The QT interval, corrected using Bazett's formula, was considered prolonged if it exceeded 440 ms, consistent with prior studies in Asian and general populations using Bazett's formula. This threshold has been widely adopted in epidemiological studies, including those in Chinese populations, due to its association with increased cardiovascular risk. However, some international guidelines recommend sex-specific thresholds (Exceeding 450 ms in men or 460 ms in women) for diagnosing long QT syndrome. The 440 ms cutoff was chosen to align with regional data and facilitate comparison with previous studies. And we set QTc > 500 ms for an extremely long QTc interval. For a short QTc interval, we used four criteria definitions to determine the prevalence and correlates (American Heart Association (AHA) criteria, <390 ms; European Society of Cardiology (ESC) criteria, <380 ms; heart rhythm, ≤330 ms; Seattle criteria, ≤320 ms) (2, 4, 19–21).

### 2.6 Statistical analysis

Descriptive statistics were calculated for all study variables, with categorical variables presented as numbers and ratios and continuous variables reported as the mean values and standard deviations. A nonparametric test or an appropriate *χ*^2^-test was used to evaluate the differences among these categories. Multivariate logistic regression analysis was employed to identify potential risk factors (age, sex, race, region, marital status, family income, medical insurance, physical activity, education, current smoking status, current drinking status, eGFR < 90 ml/m^2^, medication, obesity, hypertension, diabetes, CHD, stroke/TIA and high TC) for QTc interval prolongation or short QTc interval with odds ratios (ORs) and corresponding 95% confidence intervals (CIs). All statistical analyses were performed using SPSS version 23.0 software, with *P* values below 0.05 were deemed statistically significant.

## 3 Results

### 3.1 Baseline characteristics

This study included a total of 35,487 participants, including 13,384 males and 22,103 females. The mean QTc interval was 429.4 ± 25.1 ms overall, with no significant difference between men (429.7 ± 25.2 ms) and women (430.0 ± 22.0 ms; *P* = 0.97). The mean age was 59.92 ± 9.52 years. The majority of the population was of Han race (94.97%), married (87.63%), and had never smoked (81.51%). A total of 50.14% of the population had hypertension, 13.99% had diabetes, 28.43% had hyperlipidemia, 2.48% had coronary heart disease, 2.48% had stroke/TIA, 2.40% had eGFR < 90 ml/min/1.73 m^2^, and 16.10% had obesity. Compared to men, women were younger (59.33 vs. 60.88 years; *P* < 0.0001), had higher heart rates (75.42 vs. 74.48 bpm; *P* < 0.0001), higher TC levels (5.30 vs. 4.99 mmol/L; *P* < 0.0001) and LDL levels (3.16 vs. 2.97 mmol/L; *P* < 0.0001) and were more likely to be obese (16.79% vs. 14.97%; *P* < 0.0001). The baseline characteristics of the population according to sex are summarized in Table 1.

**Table 1:** Population characteristics according to sex.

Characteristics	Overall population (*n* = 35,487)	Male (*n* = 13,384)	Female (*n* = 22,103)	*P* value
Age, years, mean ± SD	59.92 ± 9.52	60.88 ± 9.56	59.33 ± 9.45	<0.0001
Region				<0.0001
Rural residents, *n* (%)	17,430 (51.53)	6,870 (51.33)	10,560 (47.78)	
Urban residents, *n* (%)	18,057 (48.47)	6,514 (48.67)	11,543 (52.22)	
Race, *n* (%)				0.6308
Han	33,701 (94.97)	12,720 (95.04)	20,981 (94.92)	
Non-Han	1,786 (5.03)	664 (4.96)	1,122 (5.08)	
Education level, *n* (%)				<0.0001
College and above	5,919 (16.68)	2,469 (18.45)	3,450 (15.61)	
Middle school	19,730 (55.60)	7,253 (54.19)	12,485 (56.49)	
Primary School and below	9,830 (27.70)	3,662 (27.63)	6,168 (27.91)	
Married, *n* (%)	31,098 (87.63)	12,429 (92.86	18,669 (84.46)	<0.0001
Annual household income ≥ ¥30,000, *n* (%)	21,304 (60.03)	8,385 (62.65)	12,917 (58.45)	<0.0001
Health insurance, *n* (%)				0.0878
New rural cooperative	16,381 (46.16)	6,086 (45.47)	10,295 (46.58	
Medical scheme	18,348 (51.70)	6,997 (52.28)	11,351 (51.36)	
Other	758 (2.14)	301 (2.25)	457 (2.07)	
Current drug use, *n* (%)	16,335 (46.03)	5,965 (44.57)	10,370 (46.92)	<0.0001
Sedentary lifestyle, *n* (%)	6,567 (18.50)	3,717 (27.77)	2,850 (12.89)	<0.0001
Drinking status, *n* (%)				<0.0001
Nondrinker	28,926 (81.51)	2,631 (63.96)	20,378 (92.20)	
Nonhabitual drinker	3,272 (9.22)	2,205 (16.47)	1,067 (4.83)	
Habitual drinker	3,289 (9.26)	2,631 (19.66)	658 (2.98)	
Smoking status, *n* (%)				<0.0001
Never	26,291 (74.09)	5,655 (42.25)	2,60,636 (93.36)	
Current smoker	7,233 (20.38)	6,050 (45.20)	1,183 (5.35)	
Noncurrent smoker	1,963 (5.53)	284 (1.28)	284 (1.28)	
Hypertension, *n* (%)	17,792 (50.14)	7,063 (52.77)	10,792 (48.54)	<0.0001
Diabetes mellitus, *n* (%)	4965 (13.99)	1,970 (14.72)	2,995 (13.55)	0.0021
Hyperlipidemia, *n* (%)	10,090 (28.43)	3,318 (24.79)	6,772 (30.64)	<0.0001
Coronary heart disease, *n* (%)	879 (2.48)	410 (3.06)	469 (2.12)	<0.0001
eGFR < 90 ml/min/1.73 m^2^	852 (2.40)	399 (3.15)	453 (2.16)	<0.0001
BMI ≥ 28 kg/m^2^	5,714 (16.10)	2,002 (14.96)	3,712 (16.79)	<0.0001
Stroke/TIA, *n* (%)	3,226 (9.09)	1,286 (9.61)	1,940 (8.78)	0.0083
Hear rate, bpm	75.42 ± 12.19	74.84 ± 13.97	75.77 ± 10.96	<0.0001
BMI, kg/m^2^	23.86 ± 7.80	23.70 ± 7.25	23.97 ± 8.11	0.0014
SBP, mmHg	131.49 ± 20.18	132.43 ± 19.37	130.92 ± 20.64	<0.0001
DBP, mmHg	80.57 ± 11.29	82.51 ± 11.38	79.39 ± 11.07	<0.0001
eGFR, ml/min/1.73 m^2^	106.27 ± 26.41	100.60 ± 24.21	109.70 ± 27.10	<0.0001
Total cholesterol, mmol/L	5.18 ± 1.10	4.99 ± 1.17	5.30 ± 1.04	<0.0001
LDL-C, mmol/L	3.09 ± 0.85	2.97 ± 0.83	3.16 ± 0.86	<0.0001
Serum creatinine, mmol/L	68.69 ± 31.35	79.70 ± 23.40	62.04 ± 33.28	<0.0001

Values are the mean ± SD or %. BMI, body mass index; TIA, transient ischemic attack; SBP, systolic blood pressure; DBP, diastolic blood pressure; eGFR, estimated glomerular filtration rate; LDL-C, low-density lipoprotein cholesterol.

### 3.2 QTc interval distribution in Chinese population

**Figure 2:**
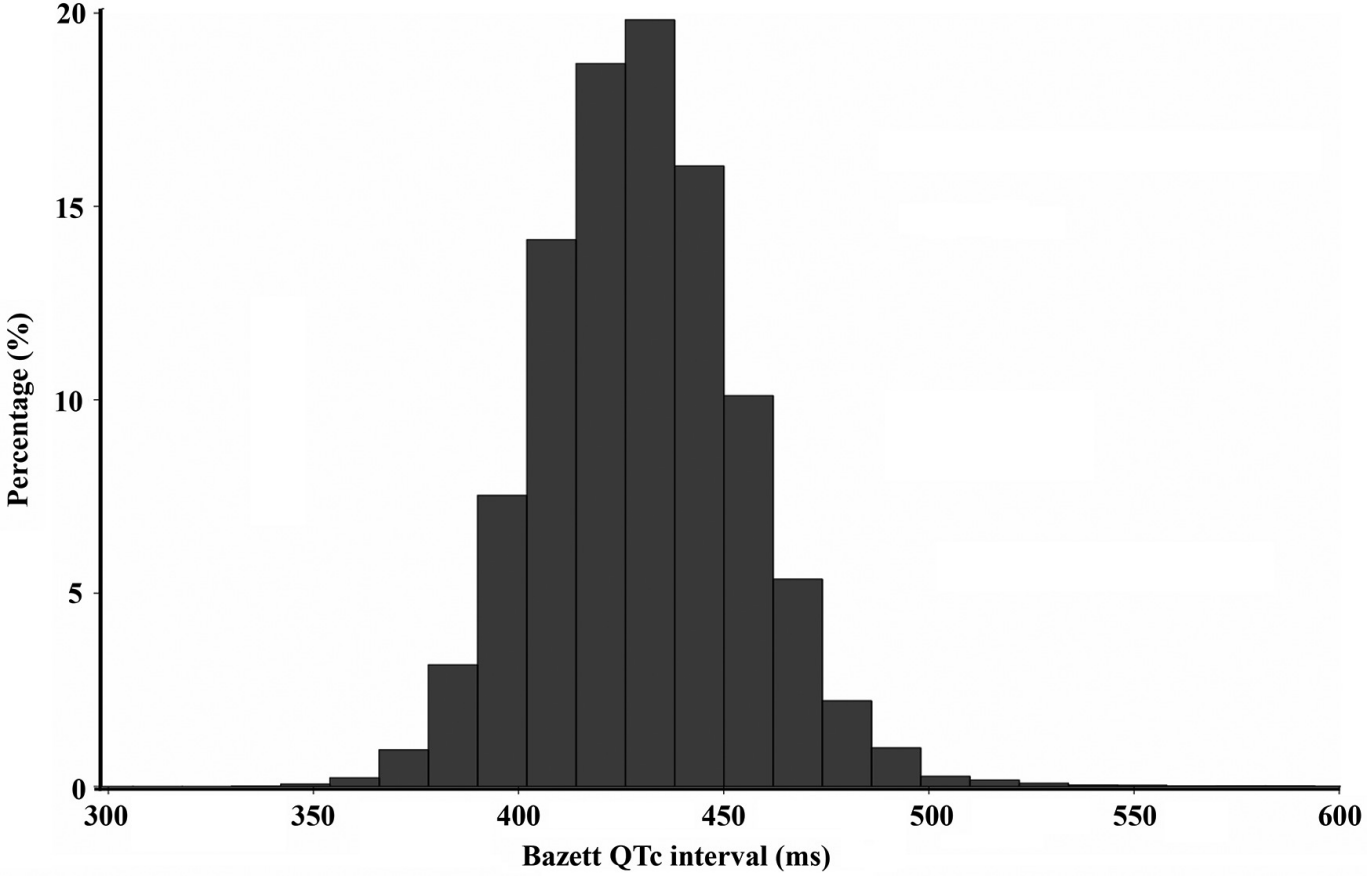
Distribution of the QTc interval in the study population.

**Figure 3:**
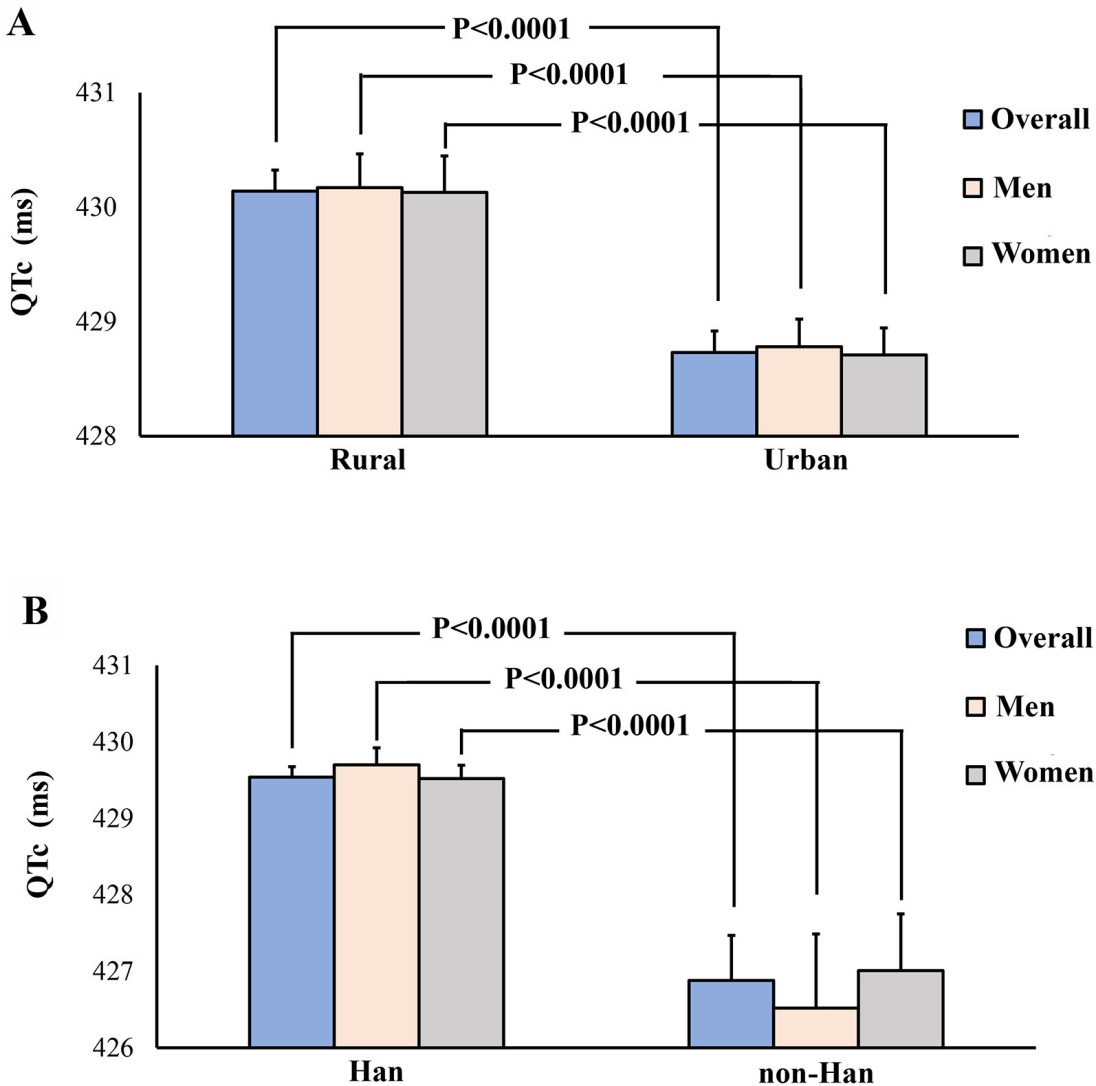
Qtc interval in different subgroups. Panel **A**: Rural regions versus urban regions; Panel **B**: Han Chinese individuals versus non-Han Chinese individuals.

The QTc intervals exhibited a normal distribution across the study population (Figure 2). The mean QT interval was 429.4 ± 25.1 ms. No significant difference was observed in the mean QT intervals between Chinese males and females (429.7 ± 25.2 vs. 430 ± 22; *P* = 0.9731). Additionally, we did not find a significant change in the QTc interval across all age and sex subgroups (45–54, 55–64, 65–74, ≥75; all *P* > 0.05) (Supplementary Table S1). Little variation was observed in the QTc interval among racial groups and different regions. Han Chinese individuals and individuals from rural regions had QTc intervals slightly longer than non-Han Chinese individuals (430.1 ± 24.8 vs. 428.7 ± 25.6; *P* < 0.0001) and individuals from urban regions (429.5 ± 25.2 vs. 426.9 ± 24.9; *P* < 0.0001) (Figure 3). Further analysis revealed that the variation between urban and rural residents was attributed to differences in the proportions of Han Chinese and non-Han Chinese populations. The proportion of Han-Chinese individuals in urban regions was 96.40%, whereas it was 93.48% in rural regions (*P* < 0.0001). One percent exhibited a Bazett-corrected QTc interval shorter than 375 ms, while 1% had a QTc interval exceeding 492 ms. The 0.5th, 1st, 2nd, and 2.5th percentiles, along with the corresponding upper percentiles, are presented in Table 2. The widely accepted definition of a normal range is based on a large population with normally distributed values, including 95% of the population. Accordingly, the normal QTc range is defined as the values between the 2.5th and 97.5th percentiles, resulting in a QTc range of 384–480 ms.

**Table 2:** Percentiles for the QT interval (bazett) in the population (*n* = 35,487).

Percentile	Mean QTc (ms)	Bazett QTc interval (ms)	
		Male	Female
0.5	370	370	370
1	375	375	375
2	382	382	382
2.5	384	384	384
5	391	391	391
95	470	470	470
97.5	480	480	479
98	483	484	482.5
99	492	492	492
99.5	505	505	505

### 3.3 Long QTc intervals and correlates

Of 35,487 participants, 11,583 and 213 qualified as cases of long QTc intervals and extremely long QTc intervals, respectively (Table 3). The prevalence of long QTc was 32.64% and 0.60%, respectively. The population characteristics are presented in Supplementary Table S2, categorized by QTc interval (QTc > 440 vs. QTc ≤ 440 ms).

**Table 3:** The prevalence of prolonged and shortened QTc intervals in the population.

QTc intervals	Overall	Men	Women	*P* value
Extreme prolonged QTc > 500 ms	213 (0.60%)	80 (0.60%)	133 (0.60%)	0.9623
Long QTc > 440 ms	11,583 (32.64%)	4,363 (32.60%)	7,220 (32.67%)	0.9623
Short QTc < 390 ms	1,727 (4.87%)	654 (4.89%)	1073 (4.85%)	0.8924
Short QTc < 380 ms	652 (1.84%)	244 (1.82%)	408 (1.85%)	0.8766
Short QTc < 360 ms	45 (0.13%)	20 (0.15%)	25 (0.11%)	0.3972
Short QTc < 330 ms	1 (0.02%)	0 (0.00%)	1 (0.03%)	0.8846

Table 4 shows the results of multivariable logistic regression analysis identifying risk factors associated with prolonged QTc interval. Han Chinese individuals exhibited a 22.8% greater risk of prolonged QTc intervals compared to non-Han Chinese individuals (OR: 1.228, 95% CI: 1.105–1.364, *P* = 0.0001). Participants from rural regions had a 59.3% increased risk for long QTc intervals (OR: 1.228, 95% CI: 1.105–1.364, *P* = 0.0001). Furthermore, participants with hypertension had 15.6% increased risks and who had medical scheme insurance coverage had 24.1% increased risks for prolonged QTc intervals (OR: 1.156, 95% CI: 1.010–1.324, *P* = 0.035; OR: 2.636, 95% CI: 1.806–3.846, *P* < 0.001).

**Table 4:** Multivariate logistic regression analyses of long QTc intervals and associated factors.

Variable	OR	(95% CI)	*P* value
Age (per 10 years)	0.996	0.969–1.023	0.7620
Sex			
Female	1.000 (reference)		
Male	1.007	0.948–1.070	0.8219
Residence			
Urban	1.000 (reference)		
Rural	1.221	1.149–1.297	<0.0001
Ethnicity			
Non-Han	1.000 (reference)		
Han	1.306	1.161–1.470	<0.0001
Education			
Primary school and below	1.000 (reference)		
Middle school and below	1.002	0.950–1.056	0.9555
College and above	1.044	0.972–1.121	0.2392
Marital status			
Unmarried	1.000 (reference)		
Married	1.023	0.951–1.101	0.5410
Health insurance			
Other	1.000 (reference)		
New rural cooperative	1.038	0.875–1.231	0.6685
Medical scheme	1.193	1.010–1.410	0.0377
Physical activity			
Sedentary lifestyle	1.000 (reference)		
Non-sedentary lifestyle	1.031	0.969–1.470	0.3312
Drug use status			
Non-current drug use	1.000 (reference)		
Current drug use	1.045	0.886–1.321	0.6873
Drinking status			
Nondrinker	1.000 (reference)		
Nonhabitual drinker	1.013	0.932–1.100	0.7673
Habitual drinker	1.080	0.993–1.175	0.0742
Smoking status			
Never	1.000 (reference)		
Current smoker	0.981	0.881–1.093	0.1786
Noncurrent smoker	0.954	0.890–1.022	0.7263
Comorbidities			
Hypertension	1.075	1.007–1.148	0.0292
Diabetes mellitus	1.055	0.987–1.129	0.1164
History of CHD	0.959	0.822–1.118	0.5918
History of stroke/TIA	0.953	0.877–1.035	0.2551
Obesity	0.996	0.896–1.041	0.9011
eGFR < 90 ml/min/1.73 m^2^	1.071	0.925–1.240	0.3598
Heart rate, bpm	1.019	0.998–1.040	0.0701
High LDL-C	0.966	0.896–1.041	0.3635

OR, odds ratios; CI, confidence interval; CHD, coronary heart disease; TIA, transient ischemic attack; eGFR, estimated glomerular filtration rate; LDL-C, low-density lipoprotein cholesterol.

### 3.4 Short QTc intervals and correlates

The study shows that the prevalence of short QT intervals was 4.87% (*n* = 1,727; AHA, <390 ms), 1.84% (*n* = 652; ESC criteria; <380 ms), or 0.02% (*n* = 1; heart rhythm, ≤330 ms), depending on the definition used (Table 3). None of the QTc intervals were less than or equal to 320 ms (*n* = 0; Seattle criteria, ≤320 ms). A total of 16 participants in our cohort had a QTc interval of ≤350 ms, including 5 males and 11 females. Using the American Heart Association criteria, which define a short QT interval as ≤380 ms, and after adjusting for all demographic variables, eGFR < 90 ml/min/m^2^ (OR = 1.617; 95% CI 1.064–2.458; *P* = 0.0463) emerged as the most significant predictor of a short QT interval (Table 5). On the other hand, obesity, hypertension, married status and rural residence reduced the risk for a short QTc interval by 21.35%, 25.6%, 30.3% and 30.7%, respectively (OR: 0.787, 95% CI: 0.621–0.996, *P* = 0.0463; OR: 0.744, 95% CI: 0.582–0.952, *P* = 0.0186; OR: 0.697, 95% CI: 0.554–0.876, *P* = 0.0020 and OR: 0.693, 95% CI: 0.560–0.857, *P* = 0.0007, respectively).

**Table 5:** Multivariate logistic regression analyses of short QTc intervals and associated factors.

Variable	OR	(95% CI)	*P* value
Age (per 10 years)	0.964	0.876–1.061	0.4512
Sex			
Female	1.000 (reference)		
Male	1.119	0.907–1.381	0.2935
Region			
Urban	1.000 (reference)		
Rural	0.693	0.560–0.857	0.0007
Ethnicity			
Non-Han	1.000 (reference)		
Han	0.768	0.541–1,091	0.1403
Education			
Primary school and below	1.000 (reference)		
Middle school and below	0.964	0.801–1.159	0.6931
College and above	0.934	0.727–1.201	0.5956
Marital status			
Unmarried	1.000 (reference)		
Married	0.697	0.554–0.876	0.0020
Health insurance			
Other	1.000 (reference)		
New rural cooperative	1,224	0.689–2.175	0.4904
Medical scheme	1.034	0.590–1.813	0.9059
Physical activity			
Sedentary lifestyle	1.000 (reference)		
Non-sedentary lifestyle	0.989	0.796–1.227	0.9173
Drug use status			
Non-current drug use	1.000 (reference)		
Current drug use	1.122	0.899–1.423	0.6573
Drinking status			
Nondrinker	1.000 (reference)		
Nonhabitual drinker	1.009	0.794–1.283	0.1786
Habitual drinker	0.755	0.499–1.142	0.7263
Smoking status			
Never	1.000 (reference)		
Current smoker	0.952	0.713–1.272	0.7413
Noncurrent smoker	0.757	0.548–1.046	0.0919
Comorbidities			
Hypertension	0.744	0.582–0.952	0.0186
Diabetes mellitus	1.033	0.819–1.304	0.7832
History of CHD	1.462	0.931–2.295	0.0991
History of stroke/TIA	1.028	0.777–1.359	0.8482
Obesity	0.787	0.621–0.996	0.0463
eGFR < 90 ml/min/1.73 m^2^	1.617	1.064–2.458	0.0245
Heart rate	1.040	0.971–1.114	0.2600
High LDL-C	0.954	0.735–1.240	0.7261

OR, odds ratios; CI, confidence interval; CHD, coronary heart disease; TIA, transient ischemic attack; eGFR, estimated glomerular filtration rate; LDL-C, low-density lipoprotein cholesterol.

## 4 Discussion

This study provides the first comprehensive epidemiological data on QTc interval distribution in Chinese adults, reporting a mean QTc of 429.7 ± 25.1 ms and a normal range of 384–480 ms. There was no notable difference in the QTc interval between Chinese adult men and women or across all age subgroups. Furthermore, the prevalence of a long QTc interval and an extremely long QTc interval was 32.64% and 0.60%, respectively. In the fully adjusted logistic regression model, Han ethnicity, rural residence, hypertension and medical scheme insurance coverage were independently associated with an increased risk for a long QTc interval (all *P* < 0.05). Moreover, the prevalence of short QT intervals was 4.87% (AHA), 1.84% (ESC) and 0.02% (heart rhythm criteria). Abnormal renal function with eGFR < 90 ml/min/m^2^ was independently associated with an increased risk for a short QTc interval.

The normal ranges reported in various studies differ to some degree, influenced by the study population's characteristics, in particular, the specific QT-adjustment formula applied. Defining normal ranges using the upper and lower bounds of the actual percentile distributions of rate-corrected QT intervals is preferable to using mean values ±2 standard deviations, as these distributions exhibit significant skewness (22, 23). We reported that the 2.5 and 97.5 percentiles of the QTc interval in the general Chinese adult population (age ≥ 45 years) were 384 and 480 ms, respectively. The upper and lower limits in this normal population were much higher than those from the Western population, suggesting a different normal range of the QTc interval, which should be considered in clinical practice (24).

Sex and age differences were two common phenomena noted in the QTc interval (7, 25). Thus, separate sex- and age-specific QT adjustment formulas have been proposed to accommodate these differences (22, 23). However, Our study did not identify differences in QTc intervals based on sex or age. A possible explanation was that the age in our population was older than that in previous studies (the mean age for our population was 60). It is reported that the sex difference appears during adolescence as a testosterone effect and diminishes after age 40 and becomes nearly negligible in older men and women.

Consistent with a previous study from China, our findings indicate that the overall prevalence of QTc prolongation was 32.64% with a 440 ms threshold defined by Bazett's formula, which was the same criteria as that used in a previous study (26). The prevalence of a long QTc interval was higher than that observed in data from other populations (27, 28). Using the same criteria, the prevalence of a long QTc interval in the Hawaiian population was found to be 21.2%, and it was approximately 7.5% among males and 16.5% among females in a Japanese cohort (27, 28). However, the prevalence of a long QTc interval was 32.64% for men and 32.67% for women in our study. Variations in geographic regions and ethnic backgrounds may account for the inconsistent findings. Furthermore, considering the year these studies were conducted, the incidence of cardiovascular disease-related factors, such as diabetes mellitus, hypertension and obesity, was higher in the present study. A prolonged QTc interval was strongly associated with increased risks of cardiovascular mortality, coronary heart disease death, sudden cardiac death and all-cause mortality (4, 29). thus, the high prevalence of prolonged QTc in the Chinese population suggests a great challenge for cardiovascular disease prevention in rural China.

The prevalence of extremely long QTc in our study was 0.6%. A similar result was found in the Jackson Heart Study, which involved an African American cohort in Mississippi. The prevalence of extreme QTcB (≥500 ms) is 0.4% and 0.5% in female and male, respectively. The study indicated that regional and racial differences, as well as differences in cardiometabolic conditions and lifestyle factors, have minimal impact on the occurrence of significantly prolonged QTc intervals (8).

Our study verified the association between hypertension and prolonged QTc intervals, aligning with prior research findings (30). Hypertension was associated with a significantly increased risk of prolonged QTc intervals, suggesting the need for a screening electrocardiogram prior to initiating medication in these groups.

The studies indicated that coronary heart disease, diabetes, and stroke are associated with an prolonged QTc interval (28, 31). However, our study didn't find significant correlations between CHD, diabetes and stroke and a prolonged QTc interval. A possible reason was that the characteristics of the selected subjects were different. The population in our study was apparently healthy. However, in those studies, the subjects were at high risk of cardiovascular disease, even the patients in hospitals.

China is a multiethnic country, and the majority are of Han ethnicity. Ethnicity modifies the association between numerous electrocardiographic abnormalities and cardiovascular disease outcomes (7, 25). In our study, we found that compared with non-Han ethnicity, the QTc interval in those of Han ethnicity is significantly longer and closely related to the risk of long QTc interval. This is the first study to describe the relationship between ethnicity and the QTc interval in China. The regional differences and correlations in the QTc interval in our study were attributed to the effect of ethnicity differences in this region. The causes of these ethnic differences remain unclear. As far as the we are aware, there are no studies reporting the mechanism between this ethnic difference, which warrants further investigation.

Medication use has a huge effect on the QTc interval (32). The proportion of medication users in our study was large. Approximately 46% of participants were used medication. However, we did not find a significant change in the QTc interval in our study. This may be attributed to the diversity of the medicine used. As described previously, the majority of medication used was cardiovascular drugs, which have less effect on the QTc interval (16).

While extensive research exists on prolonged QT intervals, there is limited knowledge about shortened QT intervals, particularly at the population level. Short QT syndrome is an uncommon disease characterized by accelerated ventricular repolarization (QT intervals generally ≤320 ms) and a heightened risk of sudden cardiac death (5, 11). The AHA, ESC and Seattle criteria define a short QT interval as <390, <380 and ≤320 ms, respectively. No of individuals with QTc ≤320 ms were detected in this population. The prevalence of a short QTc ≤330 ms in this population was 0.02%, and the incidence of a short QTc ≤380 ms was 1.84%. This figure was significantly lower than that observed in Swiss adults and a Japanese population cohort with a mean age of 53 ± 20 years (33, 34). One potential reason for the absence of participants with a Bazett-corrected QTc interval ≤320 ms in our study could be that individuals with such short QTc intervals may not survive into adulthood. Using the definition of ESC criteria, our study found that abnormal renal function was related to the risk of short QTc intervals, while obesity, hypertension, marital status and rural residence were conversely related to short QTc intervals. Thus, it is speculated that the lower prevalence of a short QTc interval in this study may be attributed to changes in life patterns. Notably, recent research demonstrates that the choice of QTc correction formula significantly impacts the measured prevalence of short QT intervals and diagnostic classification of Short QT Syndrome (SQTS). For instance, the Framingham formula identifies more individuals with QTc ≤330 ms compared to the Bazett formula in young adult cohorts, suggesting methodological variations may partly explain discrepancies across studies (35). In the reported cases of short QT syndrome and cardiac arrest, the QTc was generally less than 300 ms. Considering that no case with a QTc Bazett ≤320 ms was found in the present population, it could be concluded that short QTc syndrome was rare in the general Chinese adult population.

An advantage of the this study was that we provided a comprehensive epidemiologic investagation of the QTc interval distribution from a large sample of the general population in China for the first time. Our study has several limitations. Although cities and provinces were randomly chosen from seven regions, the selection of communities and villages within them was based on practical considerations, with subsequent units sampled according to population census-based weights. Consequently, our study may be subject to some sampling bias and potentially compounded by participation bias, given the 75% response rate; participants were likely healthier and may have differed in other characteristics from non-respondents or those who declined participation. Second, our study has a cross-sectional design, which limited our ability to infer causal associations and to investigate clinical outcomes (e.g., major adverse cardiovascular events) with longitudinal follow-up. Third, Our study may be constrained by residual confounding from both known and unmeasured factors influencing QT duration. For instance, we did not assess electrolyte levels, such as potassium or magnesium, which are recognized to impact QT intervals (36, 37). Finally, QT interval abnormalities are closely related to inherited disease (38, 39). The absence of genetic data is a huge limitation that restricts further research into the pathogenesis of clinical disease in the present population. However, as a population-based study, our findings are applicable to the broader Chinese population and offer critical insights into the distribution of QTc intervals in China. Furthermore, the stability of our population-level inferences is enhanced by conducting our study within a comprehensive census that encompasses nearly the entire study population.

## 5 Conclusion

In conclusion, previous epidemiological data on ECG abnormalities, including QT abnormalities, and their related factors has predominantly relied on studies performed in Western populations. Our large national survey showed that the mean Bazett QTc interval was 429.4 ± 25.1 ms. The normal range for the QTc interval was 384–480 ms. Furthermore, the prevalence of long QTc intervals and extremely long QTc intervals was 32.64% and 0.60%, respectively. In the fully adjusted logistic regression model, Han ethnicity, rural residence, hypertension and medical scheme insurance coverage were independently associated with an increased risk for a long QTc interval (all *P* < 0.05). Moreover, the prevalence of short QT intervals was 4.87% (American Heart Association criteria), 1.84% (European Society of Cardiology criteria) and 0.02% (Heart Rhythm criteria). An eGFR < 90 ml/min/m^2^ was independently associated with an increased risk for a short QTc interval. Considering the substantial and rapidly evolving demographic characteristics and lifestyle patterns of the Chinese population, our findings of a higher prevalence of long QTc intervals raise concerns regarding health policy and practice in this country.

## Data Availability

The raw data supporting the conclusions of this article will be made available by the authors, without undue reservation.
