# Supplementary material for: Population prevalence and correlates of prolonged and shortened QTc intervals in a nationwide survey of adults in China: a report from Chinese arrhythmia epidemiology cross-sectional study
Source: Front Cardiovasc Med. 2025 Jul 31;12:1555512. doi: 10.3389/fcvm.2025.1555512 (PMC12350289; doi:10.3389/fcvm.2025.1555512)
Supplement: Supplementary file 1 [file Table1.pdf]

Supplementary Table 1. Characteristics by QTc interval (QTc >440ms Vs. QTc ≤440ms).

	QTc Bazett <440ms (n=23904)	QTc Bazett ≥440ms (n=11583)	P value
Age, years	59.92±9.56	59.90±9.44	0.8687
**Sex**			0.8968
Female	14883(62.26%)	7220(62.33%)	
Male	9021(37.74%)	4363(37.67%)	
**Residence**			0.0003
Rural	12323(51.55%)	5734(49.50%)	
urban	11581(48.45%)	5849(50.50%)	
**Ethnicity**			0.0001
Non-Han	1277(5.34%)	509(4.39%)	
Han	22627(94.66%)	11074(95.61%)	
**Education level**			0.5118
Primary School or below	6637(27.77%)	3193(27.57%)	
Middle School	13318(55.71%)	6420(55.43%)	
College or above	3949(16.52%)	1970(17.01%)	
**Marital status**			0.5922
Married	20932(87.57%)	10166(87.77%)	
Un-married	2972(12.43%)	1417(12.23%)	
Health insurance, n (%)			0.0735
Other	528(2.21%)	230(1.99%)	
New Rural Cooperative	11106(46.46%)	5275(45.54%)	
Medical Scheme	12270(51.33%)	6078(52.47%)	
**Physical activity**			0.7600
Sedentary lifestyle	4434(18.55%)	2133(18.41%)	
Un- Sedentary lifestyle	19470(81.45%)	9450(81.59%)	
**Drinking Status**			

Non-drinker	19510(81.62%)	9416(81.29%)	0.5156
Nonhabitual drinker	2208(9.24%)	1064(9.19%)	
Habitual drinker	2186(9.14%)	1103(9.52%)	
**Smoking status**			0.5376
Never	17669(73.92%)	8622(74.44%)	
Current smoker	4911(20.54%)	2322(20.05%)	
Noncurrent smoker	1324(5.54%)	639(5.52%)	
**Marital status**			0.5922
Married	20932(87.57%)	10166(87.77%)	
Un-married	2972(12.43%)	1417(12.23%)	
**Economic level**			0.0001
⩾¥30000	14185(59.34%)	7119(61.46%)	
<¥30000	9719(40.66%)	4464(38.54%)	
**Complication**			
Hypertension	12008(50.23%)	5784(49.94%)	0.5973
Diabetes Mellitus	3294(13.78%)	1671(14.43%)	0.0999
Hyperlipidemia	6745(28.22%)	3345(28.88%)	0.1952
Coronary heart disease	600(2.51%)	279(2.41%)	0.5647
Stroke/TIA	2197(9.19%)	1029(8.88%)	0.3452
obesity	3878(16.22%)	1836(15.85%)	0.3708
<90 mL/min/ 1.73m^2	563(2.49%)	289(2.64%)	0.4110
BMI, kg/m^2	23.88±8.28	23.84±6.69	0.6490
SBP, mmHg	131.68±20.36	131.10±19.79	0.0112
DBP, mmHg	80.68±11.37	80.34±11.12	0.0072
Heart rate, bpm	75.31±11.25	75.64±13.93	0.0268
eGFR, mL/min/ 1.73m^2	106.11±26.29	106.62±26.67	0.9300
Total cholesterol, mmol/l	5.18±1.11	5.18±1.11	0.9300
LDL cholesterol, mmol/l	3.10±0.86	3.08±0.84	0.0772
